# Current Status of Biomarkers in Anti-N-Methyl-D-Aspartate Receptor Encephalitis

**DOI:** 10.3390/ijms222313127

**Published:** 2021-12-04

**Authors:** Nicolás Lundahl Ciano-Petersen, Pablo Cabezudo-García, Sergio Muñiz-Castrillo, Jérôme Honnorat, Pedro Jesús Serrano-Castro, Begoña Oliver-Martos

**Affiliations:** 1 Neuroimmunology and Neuroinflammation Group, Biomedical Research Institute of Málaga (IBIMA), 29007 Málaga, Spain; lundahl151@gmail.com (N.L.C.-P.); pablocabezudo@gmail.com (P.C.-G.); 2 Red Andaluza de Investigación Clínica y Traslacional en Neurología (Neuro-RECA), 29010 Málaga, Spain; 3 French Reference Center on Paraneoplastic Neurological Syndromes and Autoimmune Encephalitis, Hospices Civils de Lyon, Hôpital Neurologique, 69677 Bron, France; sermucas@gmail.com (S.M.-C.); jerome.honnorat@chu-lyon.fr (J.H.); 4 SynatAc Team, Institut NeuroMyoGène, INSERM U1217/CNRS UMR 5310, Université de Lyon, Université Claude Bernard Lyon 1, 69372 Lyon, France; 5 Department of Cell Biology, Genetics and Physiology, Physiology Area, University of Malaga, 29010 Málaga, Spain

**Keywords:** anti-NMDAR encephalitis, biomarker, rare diseases, autoimmune encephalitis

## Abstract

The discovery of biomarkers in rare diseases is of paramount importance to allow a better diagnosis, improve predictions of outcomes, and prompt the development of new treatments. Anti-N-methyl-D-aspartate receptor (NMDAR) encephalitis is a rare autoimmune disorder associated with the presence of antibodies targeting the GluN1 subunit of the NMDAR. Since it was discovered in 2007, large efforts have been made towards the identification of clinical, paraclinical, and molecular biomarkers to better understand the immune mechanisms that govern the course of the disease as well as to define predictors of treatment response and long-term outcomes. However, most of these biomarkers are still in an exploratory phase, with only a few candidates reaching the final phases of the always-complex process of biomarker development, mainly due to the low incidence of the disease and its recent description. Clinical and paraclinical markers are probably the most widely explored in anti-NMDAR encephalitis, five of them combined in a clinical score to predict 1 year outcome. On the contrary, soluble molecules, such as persistent antibody positivity, antibody titers, cytokines, and other inflammatory mediators, have been proposed as biomarkers of clinical activity, inflammation, prognosis, and treatment response, but further studies are required for their clinical validation including larger and more homogenous cohorts of patients. Similarly, genetic susceptibility biomarkers are still in the exploratory phase and, therefore, weak conclusions can for now only be achieved. Thus, further studies are warranted to define biomarkers and unravel the underlying mechanisms driving rare diseases such as anti-NMDAR encephalitis. Future international collaborative studies with prospective designs that enable the enrollment of large cohorts will allow for the identification and validation of novel biomarkers for clinical decision-making.

## 1. Introduction

Anti-N-methyl-D-aspartate receptor (NMDAR) encephalitis is a rare autoimmune disorder associated with antibodies targeting the GluN1 subunit of the NMDAR. Despite being the most frequent autoimmune encephalitis (AE) in adults and the second in children, it is still considered a rare disease (ORPHA: 217253) with an estimated incidence of 1 case per million population per year [1,2,3].

The definition of rare disease varies depending on the region that is being assessed. While in Europe it is defined as a disease affecting less than 1/2000 people, in the United States it has to affect less than 200,000 Americans, giving a threshold of approximately 1/1650 people, considering the current population [4]. These diseases usually have unmet medical needs, since the development of clinical trials for novel drugs presents multiple challenges due to the common geographic dispersion of patients, phenotypic heterogeneity, and poor understanding of the pathophysiology [5]. Therefore, the development of biomarkers in rare diseases is a pressing need that will likely contribute to the understanding of their pathogenesis and provide practical tools for the diagnosis, outcome prediction, and development of novel treatments.

A biomarker is defined as an objectively measurable characteristic evaluated as an indicator of physiological functions, pathogenic processes, and responses to an exposure or intervention [6,7]. However, not all biomarkers are suitable for daily clinical practice, and the ideal biomarker is considered to be disease-specific, cost-effective, minimally invasive, reproducible with adequate sensitivity and specificity, and to correlate with disease outcomes [8,9]. Moreover, in the evaluation process of a potential candidate, the general framework for developing disease-related biomarkers comprises different steps including biomarker discovery, analytical validation, qualification, and establishment of clinical utility [10]. According to their applicability, biomarkers can be further classified as prognostic, susceptibility/risk, diagnostic, safety, monitoring, and predictive and treatment-response biomarkers [11].

However, due to the aforementioned limitations, the only biomarker widely used in clinical practice in anti-NMDAR encephalitis is the identification of IgG antibodies against the GluN1 subunit of the NMDAR in the cerebrospinal fluid (CSF), which are mandatory to achieve a definite diagnosis [2]. Thus, no other biomarker is used for diagnostic, prognostic, monitoring, or therapeutic guidance besides clinical and paraclinical scores [12]. Given this situation, the description of novel biomarkers in anti-NMDAR encephalitis is essential, especially to improve the current therapeutic management and to promote the development of new treatments that might accelerate recovery [2].

**Figure 1:**
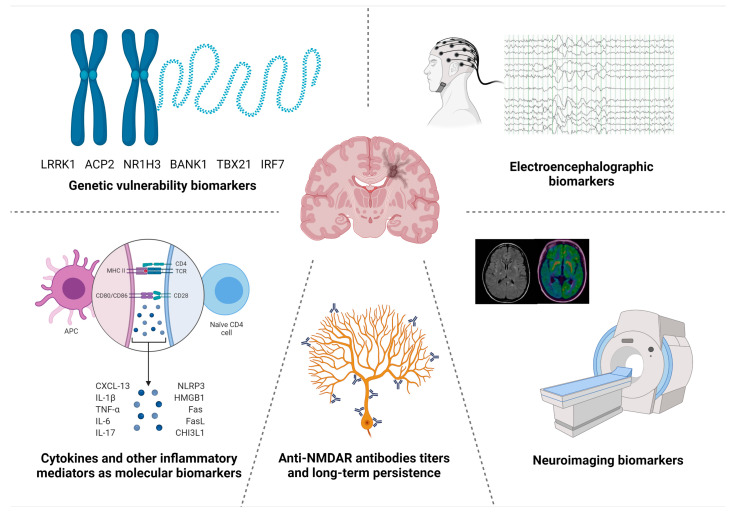
Overview of paraclinical and molecular biomarkers proposed in anti-NMDAR encephalitis.

In this review, we present an overview of the biomarkers described so far in anti-NMDAR encephalitis, from clinical and paraclinical features identified during the routine diagnostic workup, to advanced molecular biomarkers that could improve our understanding of anti-NMDAR encephalitis pathophysiology and lead to the development of novel targeted-treatments (Figure 1).

## 2. An Overview of Anti-NMDAR Encephalitis

Anti-NMDAR encephalitis is a rare autoimmune neurological disorder that predominantly affects women (≈80%) in the first four decades of their life. This AE is caused by autoantibodies targeting the NMDAR, impairing synaptic glutamatergic networks involved in brain circuits essential for learning, memory, and neuroplasticity [13]. The majority of patients initially present subacute psychiatric symptoms such as mania, social withdrawal, and psychosis. Subsequently, this initial clinical picture is rapidly followed by neurological abnormalities including short-term memory impairment, seizures, movement disorders, central hypoventilation, and even altered level of consciousness leading to intensive care unit (ICU) in up to 50% of adult patients [14]. Nonetheless, movement disorders and partial seizures are more frequently the first symptom in children [15,16], while male adults often have isolated focal seizures preceding the full clinical picture [17]. Interestingly, up to 70% of patients report prodromal flu-like symptoms, suggesting a potential role of yet unknown environmental/infectious agents that might act as triggers of the aberrant immune response [18].

Initially, anti-NMDAR encephalitis was described as a paraneoplastic neurological syndrome (PNS) associated with ovarian teratomas that aberrantly express neural antigens, which lead to an immune cross-reaction against the central nervous system [19]. Supporting this hypothesis, Chefdeville et al. found that ovarian teratomas associated with anti-NMDAR encephalitis contain nervous glial tissue, and among them, 82% express the GluN1 subunit of the NMDAR [20]. However, a recent meta-analysis showed that paraneoplastic cases account for only 25% of cases [14], suggesting that other unknown pathogenic mechanisms may lead to the immune tolerance breakdown in anti-NMDAR encephalitis. Likewise, a few viral infections have been reported as triggers of anti-NMDAR encephalitis, especially herpes simplex 1 virus (HSV1) encephalitis [13,21,22]. A prospective study of patients with HSV1 encephalitis found that 27% of them subsequently developed an AE after a mean latency of 32 days, and, strikingly, all these patients presented CSF antibodies against neuronal antigens, 64% of them against NMDAR [22]. The underlying pathogenic mechanisms of post-infectious autoimmune encephalitis are still obscure, but several hypotheses have been proposed [21,22,23]. For instance, mechanisms involving molecular mimicry and chronic polyclonal expansions have been proposed based on serological studies [24,25], although this possibility would not explain the wide variety of antibodies against neural antigens reported, neither the fact that other infections, such as Japanese encephalitis, have also been associated with post-infectious autoimmune encephalitis [21,22,23]. Conversely, HSV1 encephalitis is typically associated with intense inflammatory and necrotic lesions involving the temporal lobes that could eventually release multiple neural antigens and trigger an aberrant self-direct immune response [23]. It is noteworthy that post-infectious autoimmune encephalitis, even with NMDAR antibodies, likely represents a different entity from idiopathic or teratoma-associated anti-NMDAR encephalitis, since the latter frequently presents better long-term outcomes [22]. However, the mechanisms behind the worse prognosis of post-herpetic anti-NMDAR encephalitis remains unclear, and the involvement of T-cell or complement-mediated cytotoxicity due to the disruption of the blood–brain barrier have been proposed [2,22].

The diagnosis of anti-NMDAR encephalitis can be achieved based on the criteria settled by Graus et al. in 2016 (Table 1), which rely on clinical criteria to reach a probable diagnosis and requires the identification of IgG NMDAR antibodies in the CSF for the definite diagnosis [26]. Only IgG antibodies have been proved to be pathogenic in vitro and in vivo and are therefore responsible for this fairly stereotyped syndrome [27,28,29]. These IgG antibodies target the GluN1 subunit of NMDAR and lead to its reversible internalization and disruption of the interaction with Ephrin B2 receptors [13,30]. The pathogenic role of these antibodies and the presence of CD19+ B cells and CD19+ and CD138+ plasma cells in the CSF suggest that humoral immunity is the major effector in anti-NMDAR encephalitis [31,32,33]. Nevertheless, histopathological studies showed not only B and plasma cell parenchymal infiltrates, but also CD3+ T cells were identified, suggesting that cellular immunity may play a role as well. However, there is no evidence of neuronal loss nor deposition of complement or antibodies, which in addition to the reversible effects of NMDAR antibodies, may explain its generally good response to immunotherapy [34,35,36].

The management of these patients is based on two main strategies. On the one hand, if a tumor is found, its removal is mandatory and should be promptly performed in order to decrease the antigenic exposure to the immune system. On the other hand, immunotherapy must also be prescribed as soon as there is a suspicion of AE [26]. Immunotherapy for AE generally comprises a two-step approach: The so-called first-line therapies, such as intravenous immunoglobulins, steroids, or plasma exchange, can be administered individually or in combination. Eventually, second-line therapies, such as rituximab and cyclophosphamide, might be considered for patients not showing a significant improvement in the first two weeks [18]. After these therapies, up to 75–80% of patients achieve good outcomes and reassume their daily activities. However, refractory patients may benefit from alternative drugs, such as tocilizumab, bortezomib, or daratumumab, which could be considered based on the effectiveness reported in small case series [37,38,39,40,41].

## 3. Clinical and Paraclinical Features as Markers of Anti-NMDAR Encephalitis

### 3.1. Clinical Predictors

The most explored subset of outcome predictors in anti-NMDAR encephalitis are based on the initial clinical characterization of this entity in large cohorts. For instance, ages younger than 2 years or older than 65 years and the need of ICU admission have been associated with poor long-term outcomes [14,15,42]. Regarding the impact of current treatments, poor outcomes have also been associated with a lack of immunotherapy in the first month after onset, while early instauration of plasma exchange, steroids with intravenous immunoglobulins, or the combination of the three has been associated with good prognosis [14]. Interestingly, treatment delay longer than 1 month and ICU admission have been combined with other immunologic and clinical variables (CSF pleocytosis > 20 cells/mm^3^, abnormal magnetic resonance imaging (MRI), and lack of improvement after 1 month from treatment onset) to predict outcomes in a grading score termed the anti-NMDAR encephalitis one-year functional status (NEOS) score [12]. Indeed, the accuracy of this score has recently been validated in the aforementioned meta-analysis including 694 patients, although it was modified due to the frequent unavailability of the time to response from treatment onset [14].

### 3.2. Neuroimaging Biomarkers

The development of neuroimaging biomarkers is an interesting field in anti-NMDAR encephalitis, since MRI is easily and widely available, even in non-research or specialized centers. The analysis of large cohorts of patients showed that only 25–50% of the patients had an abnormal MRI during the acute phase of the disease. However, the characterization of specific patterns is challenging due to the heterogeneity of the findings, mainly T2/FLAIR hyperintensities in temporal and frontal lobes and, in some cases, demyelinating lesions [14,15,18,43,44]. Interestingly, the sole presence of MRI abnormalities in the acute phase was found to be an independent predictor of poor outcome [12,45]. Furthermore, although most of these inflammatory changes tend to disappear during the course of the disease, some patients subsequently develop hippocampal, cerebellar, or diffuse brain atrophy [46,47]. While diffuse brain atrophy has been related to a severe acute phase, it showed no association with long-term outcomes, as it may be reversible. Conversely, cerebellar atrophy was suggested to be irreversible and, therefore, associated with poor long-term outcomes [48]. Moreover, advanced multimodal structural imaging and functional MRI studies found impaired hippocampal connectivity and white matter changes despite having normal baseline MRIs, which correlated with disease severity and memory performance [49,50].

In contrast, positron emission tomography (PET) with 18F-fluorodeoxyglucose (18F-FDG) imaging is less available, mainly due to the fact of technical and economic limitations. Similar to MRI findings, 18F-FDG abnormalities are overall heterogeneous, although a distinct pattern with mild frontal and temporal hypermetabolism and occipital hypometabolism have been described in small series of patients with anti-NMDAR encephalitis, which was associated with disease severity and improved in the recovery phase [46,51]. Interestingly, this characteristic pattern has been proposed to be related to the density of NMDAR in the brain cortex, which shows a postero–anterior gradient, opposed to the antero–posterior gradient described with 18F-FDG PET [52].

Neuroimaging biomarkers fulfill several criteria for an ideal biomarker, as they are minimally invasive, cost-effective, reproducible, and may be associated with outcomes and disease severity, although most findings are not specific to anti-NMDAR encephalitis. However, 18F-FDG PET abnormalities may be found in patients with normal MRI, highlighting its potential value as a prognostic biomarker [8,9,46].

### 3.3. Electroencephalographic Biomarkers

The role of electroencephalography (EEG) in anti-NMDAR encephalitis has been assessed in large cohorts of patients, mainly due to the fact of its availability and non-invasive nature. Interestingly, EEG abnormalities are considered a consequence of the antibody-mediated disruption of NMDAR synaptic functions [53]. Since they are found in 80–90% of patients, they were included in the diagnostic criteria of probable anti-NMDAR encephalitis [14,15,26,54].

EEG abnormalities associated with encephalopathy (mainly slowing, including delta range slowing) are the most frequent finding (≈60%), followed by epileptiform discharges and electric seizures, detected in 15% and 10%, respectively [54]. Although most of these EEG findings are not specific, a characteristic pattern defined as extreme delta-brush (EDB), consisting of a generalized rhythmic delta activity with a superimposed rhythmic beta activity, have been particularly associated with anti-NMDAR encephalitis [55].

The development of EEG abnormalities during the course of the disease seems to be time dependent, with predominant epileptiform discharges in early phases and generalized slowing predominantly in late stages [43]. Among non-epileptic abnormalities, three EEG patterns have been described in the following chronological order: excessive beta activity range 14–20 Hz (EBA) in 71% of patients, EDB in 58%, and generalized rhythmic delta activity (GRDA) in 50% [56], although lower frequencies were found in other studies [54].

The identification of a normal posterior rhythm in the first EEG performed has been associated with good outcomes [57], which may reflect normal electroencephalographic activity in the cortical areas with the highest NMDAR density in contrast to the FDG-PET pattern that associated a posterior hypometabolism with a more severe disease [51]. To the contrary, the sole presence of EEG abnormalities has not been independently associated with poor outcomes, probably due to the fact of their identification in the majority of patients [12]. However, several specific EEG patterns have been proposed as prognostic biomarkers. Among them, EDB has been associated with longer hospitalization, ICU admission, and poor long-term outcomes [14,55,58], while GRDA was strongly associated with abnormal movements [56] and treatment-response to second-line therapies [59]. In contrast, EEG findings in long-term monitoring during the acute phase of the disease have not been found to predict the persistence of seizures in long-term follow up [60].

Additionally, EEG findings can be measured and analyzed in terms of frequency, amplitude, power, and rhythmicity by different quantitative EEG techniques that have shown promising results in identifying several patterns or parameters proposed as diagnostic and prognostic biomarkers. For instance, an increased beta/delta power ratio have been associated with anti-NMDAR encephalitis compared to other AE [61], while a high-frequency peak in the delta band and a wide parietal amplitude-integrated EEG band have been associated with a poor prognosis [62,63].

Electroencephalographic findings fulfill some of the requirements for an ideal biomarker, since they are cost-effective, non-invasive, reproducible, and may be associated with prognosis [8,9]. However, although several patterns are considered to be highly suggestive of anti-NMDAR encephalitis, none has been proved to be disease specific and must be studied in larger cohorts of prospective patients.

## 4. Molecular Biomarkers in Anti-NMDAR Encephalitis

Two different approaches can be followed in the assessment of new molecular candidates for the discovery of novel biomarkers. On the one hand, a deductive reasoning based on the existing knowledge of the pathophysiology of similar disorders could be extrapolated to the disease of interest. On the other hand, an omics-based investigation is an undirected approach that allows a wider understanding of the disease and the discovery of new candidates not previously explored, although its cost and complex analysis may limit its use [64]. However, despite significant investments in biomarker discovery studies, only a small proportion of the initially proposed biomarkers are subsequently accepted and implemented in clinical practice [65].

Studies assessing potential molecular biomarkers in anti-NMDAR encephalitis are scarce, mainly due to the low incidence of the disease and its recent description in 2007 [1,19]. While clinical and paraclinical biomarkers have been assessed in large cohorts of patients and may soon be used to guide clinical practice, most molecular candidates are still in the initial phase of the biomarker development process. Most of the exploratory biomarker studies conducted in anti-NMDAR encephalitis follow a deductive reasoning to identify candidates based on existing knowledge of the pathophysiology of other autoimmune disorders. On the contrary, to our knowledge, there are no studies following an unbiased strategy employing omics to identify candidate biomarkers on the basis of their differential expression between healthy controls, other neuroimmune disorders, and patients with anti-NMDAR encephalitis.

### 4.1. Blood and CSF Soluble Biomarkers

#### 4.1.1. Anti-NMDAR Antibodies

CSF NMDAR antibodies of the IgG subclass are the most important diagnostic biomarker, as they are highly specific to the stereotyped clinical syndrome defining anti-NMDAR encephalitis [18,27,66]. These antibodies can also be found in serum, although the risk of false-positive and false-negative diagnoses should be considered if CSF is not tested as well [67]. Antibodies of other subclasses, such as IgA or IgM, have been found in patients with other neurological disorders, and IgA NMDAR antibodies have been proposed as a biomarker of teratoma-related anti-NMDAR encephalitis [68]. However, IgA or IgM NMDAR antibodies have been found to bind a different epitope and, therefore, they are unable to significantly alter the density of NMDAR clusters in cultured live neurons, questioning their role in the pathogenesis of anti-NMDAR encephalitis [66].

**Figure 2:**
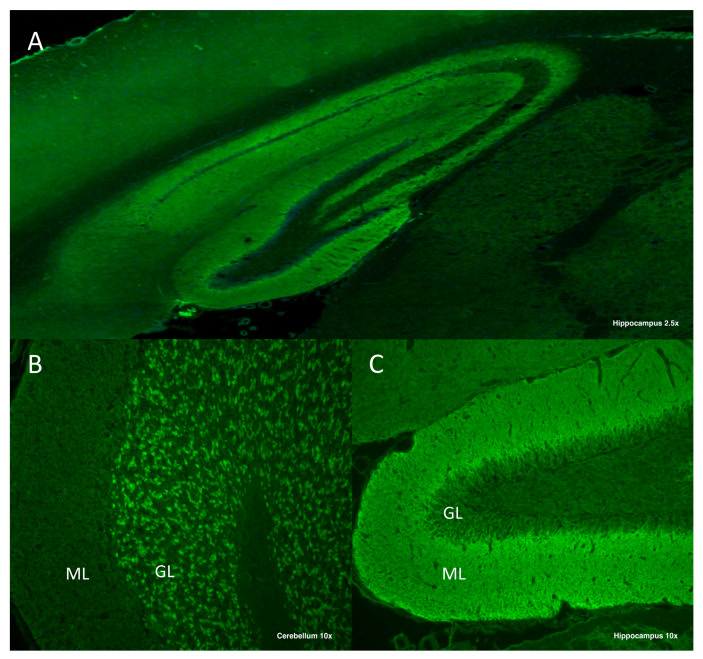
Immunostaining of an adult rat’s brain tissue with CSF (1:10) of a patient with anti-NMDAR encephalitis. A strong reactivity can be observed in the molecular layer (ML) of the hippocampus (**A**) and the granular layer (GL) of the cerebellum (**B**). A predominant reactivity with the inner part of the ML in the dentate gyrus is considered a highly suggestive pattern of anti-NMDAR antibodies (**C**).

For NMDAR antibodies detection, most clinical laboratories use a commercial test (FA112d-1005-51 or FA112d-1010-51) based on a cell-based assay (CBA) using human embryonic kidney (HEK) cells transfected with a cDNA coding for GluN1/2, that is expressed in their membrane. This test is approved by the FDA under the label “In vitro diagnostic” (IVD) and was recently reported to successfully identify NMDAR antibodies in 98.5% of the patients after a screening with rat brain tissue immunohistochemistry compared to in-house techniques of the research center that discovered these antibodies [69]. However, CBAs may lead to false-negative or false-positive results if not confirmed with tissue-based assay, especially if only serum is tested [2,66,67]. For instance, patients with neuropsychiatric systemic lupus erythematosus may have antibodies targeting the GluN2 subunit, leading to false positive results since anti-NMDAR antibodies target the GluN1 subunit [70]. For that reason, most reference research laboratories perform a rat brain tissue immunohistochemistry or immunofluorescence with serum or CSF to identify the presence of IgG antibodies targeting neural antigens, displaying a particular staining pattern when NMDAR IgGs are present (Figure 2). Then, more specific tests, such as in-house CBAs or live neuron cultures expressing NMDAR, are required to ensure that the antibodies bound to the rat brain tissue are targeting the NMDAR. Nevertheless, although these techniques are considered to be the most sensitive and specific to detect NMDAR antibodies, they are limited by their high complexity, as they require an experienced team for the interpretation of the results [66,67].

Since NMDAR antibodies have extensively been proved to be pathogenic and they are synthetized both systemically and intrathecally, it could be expected that serum and CSF titers have an influence on clinical severity and long-term prognosis. Interestingly, in vitro experiments on rat hippocampal neuronal cultures have shown that the CSF of patients with high antibody titers produced a higher decrease in synaptic and extrasynaptic NMDARs [27]. However, antibody titers are not currently used in clinical practice because their value in predicting disease severity and outcomes has only been attained in small series of patients [27,43,67]. Paraneoplastic cases have been reported to have higher serum and CSF titers, suggesting a more intense inflammatory reaction in this subset of patients, despite having a better long-term prognosis compared to non-paraneoplastic cases if the tumor is removed [27,43,67]. Conversely, patients with the highest serum and CSF titers have been suggested to have a more severe disease or even to die more frequently, while patients with the lowest titers had a milder disease. Moreover, patients with a good response to immunotherapy showed a more prominent decrease in antibody titers compared to those with no improvement [27,43,67].

The persistence of NMDAR antibodies in serum and CSF despite second-line immunotherapies has been associated with poor outcomes, or even death, in small cohorts of patients [27,43]. However, their value as a biomarker of relapses in recovered patients is worthy of future analysis, as some patients have been reported to maintain intrathecal synthesis for more than a decade without evident clinical consequences [71].

The detection of NMDAR antibodies in CSF meets some of the characteristics required for an ideal biomarker, since it is biologically and pathophysiologically relevant, and it presents a sensitivity and a specificity of 100% if brain tissue immunohistochemistry and an in-house CBA is performed [8,9,67]. However, an invasive method like the lumbar puncture is required to obtain CSF and the antibody-testing methods are not accessible in many institutions [26].

#### 4.1.2. Cytokines

A better understanding of the inflammatory molecules that govern neuroinflammation may lead to the development of novel biomarkers and treatment strategies. Among those molecules, cytokines are soluble signaling proteins used by immune cells to regulate inflammatory responses in both health and disease, and an unbalanced cytokine expression is considered a hallmark of autoimmunity. Despite the fact that most of these molecules have pleiotropic effects, some cytokines are predominantly involved in the regulation of particular subsets of immune cells that may play a relevant role in the pathogenesis of anti-NMDAR encephalitis [72]. Therefore, several studies have explored the CSF cytokine profile of anti-NMDAR encephalitis; for instance, a few interleukins (ILs), tumor necrosis factors (TNFs), chemokines, and interferons (IFNs) have been associated with clinical activity, inflammation, and long-term outcomes (Table 2).

Different cytokine profiles have been observed during the course of anti-NMDAR encephalitis compared to the control without autoimmune neurological diseases. In the acute phase, several cytokines involved in the recruitment and proliferation of B cells have been found elevated in the CSF of patients, such as the chemokine C-X-C motif ligand (CXCL) 13 [73,74,75,76,77,78], B-cell activating factor of the tumor necrosis factor family (BAFF), and a proliferation-inducing ligand (APRIL) [79]. Additionally, although anti-NMDAR encephalitis is considered to be driven mainly by humoral immunity, high levels of several cytokines playing a role in T-cell recruitment and proliferation have also been found in the CSF during the acute phase of the disease such as IFN-γ [74,78,80], tumor necrosis factor (TNF)-α [74,76,78,80,84], CXCL-10 [74,76,78], chemokine C-C motif ligand (CCL) 22 [77,78], IL-1β [77,81], IL-6 [75,76,77,81,82,84], IL-7 [74,78], IL-10 [76,78,84], and IL-17A [74,75,77,81,82,83]. Conversely, only cytokines involved in the T-cell responses, such as IFN-γ, TNF-α, CXCL-10, IL-7, and IL-17A, can be found elevated in the CSF months after the acute phase of the disease, revealing a possible role of T cells in the maintenance of the immune response [74]. Interestingly, the elevated levels of these cytokines have also been correlated with clinical severity, measured with the modified Rankin score (mRS) such as CXCL-13 [76], CXCL-10 [76,78], CCL-22 [77,78], IL-6 [77,78,81,83], IL-10 [78], and IL-17A [81,83]. On the contrary, during relapses of anti-NMDAR encephalitis only CXCL-13 [73,76], CXCL-10 [76], and IL-17A [77] have been found elevated in the CSF.

As mentioned before, a CSF pleocytosis > 20 cells/mm^3^ has been shown to predict 1 year poor functional outcomes when included in a clinical score along with other clinical variables [12]. Accordingly, CSF levels of the chemokines CXCL-13 and CXCL-10, which are involved in the recruitment of B and T cells into the central nervous system (CNS), have been found to correlate with the total number of white cells counts in the CSF and with functional outcomes of patients with anti-NMDAR encephalitis, respectively [74]. Moreover, high CSF concentrations of other cytokines involved in the proliferation of B and T cells, such as BAFF, APRIL, and IL-17A, have also been associated with poor long-term outcomes [77,79].

Thus, cytokines are promising biomarkers, since they fulfill several criteria for an ideal biomarker, as they are cost-effective and correlate with disease outcomes. However, most of the aforementioned cytokines have only been studied in the CSF, requiring an invasive test for its extraction, and none of them have been proposed to be specific of anti-NMDAR encephalitis [8,9]. Although some cytokines, such as CXCL-13, IL-1β, IL-6, IL-17, and TNF-α, have been studied in relatively large cohorts of patients (*n* = 100–270) and can therefore be considered to be in the validation stage of the biomarker development process, most of the remaining were explored in small samples of patients and need further confirmatory studies.

#### 4.1.3. Other Molecular Biomarkers

Interestingly, the innate immune system could also play a role in the pathogenesis of anti-NMDAR, as it is suggested by the high CSF levels of certain proteins involved in the activation of innate responses found in small series of patients such as the NRL family pyrin domain-containing 3 (NLRP3) inflammasome, soluble Fas and FasL, and the high-mobility group box protein 1 (HMGB1) (Table 2) [81,82,87]. Moreover, the levels of NLRP3, sFas, and sFasL were reported to correlate with clinical severity during the acute and recovery phases of the disease, suggesting their potential role as prognostic biomarkers [81,86,87].

Other mediators of neuroinflammation have been found to be elevated in the serum and CSF of patients with anti-NMDAR encephalitis and to correlate with their functional status such as chitinase-3-like 1 (CHI3L1), osteopontin (OPN), pentraxin 3 (PTX3), CD40L, CD146, and CD138 (Table 2) [80,83,84,85]. Additionally, biomarkers of axonal damage, such as neurofilaments, a cytoplasmic protein highly expressed in myelinated axons, have been quantified and associated with outcomes in other neurological disorders [88]. In anti-NMDAR encephalitis, CSF levels of neurofilament light and heavy chains were correlated with the clinical severity and the levels of the cytokines IL-1β and IL-17A [89].

### 4.2. Genetic Susceptibility Biomarkers

The heterogeneity of the environmental factors described in anti-NMDAR encephalitis could potentially interplay with an eventual genetic predisposition. The human leukocyte antigen (HLA) is the main genetic factor related to autoimmunity, with several associations in neurological diseases driven by neural antibodies [90], being especially strong between DRB1*07:01 and limbic encephalitis (LE) with antibodies against leucine-rich glioma inactivated 1 [91,92,93,94] and between DRB1*11:01 and LE with antibodies against contactin-associated protein-like 2 (CASPR2) [93,95]. On the contrary, a first study including a small cohort of patients with anti-NMDAR encephalitis found no association with HLA [91]. Later, very weak and doubtful associations were reported with B*07:02 in adult patients of Caucasian origin [96] and with DRB1*16:02 in a Chinese cohort [97]. However, these associations have not been confirmed in a very recent genome-wide association study (GWAS) including the largest cohort (*n* = 178) investigated to date [98].

Since no consistent association between HLA and anti-NMDAR encephalitis has so far been found, other non-HLA genes have preliminary been explored. Nevertheless, the first GWAS performed in anti-NMDAR encephalitis obtained no positive results, although the number of patients included was fairly small [96]. The same authors have very recently doubled the sample size and reported a significant association with LRRK1 (leucine-rich repeat kinase 1), ACP2 (lysosomal acid phosphatase), and NR1H3 (nuclear receptor subfamily 1 group H member 3) genes, which might have some yet poorly defined functions in immune and inflammatory responses [98]. Despite being appealing, these results have to be carefully considered, since there was no replication cohort due to the still insufficient sample size [98].

In addition, using a different approach that consisted in genotyping a preselected list of 28 genes, a Chinese study found an association with polymorphisms in BANK1 (B-cell scaffold protein with ankyrin repeats), TBX21 (T-box transcription factor 21), and IRF7 (interferon regulatory factor 7) [99]. Moreover, IRF7 and TBX21 are involved in the immune response against viruses, which is interesting considering that nearly 70–80% of patients with anti-NMDAR encephalitis have prodromal flu-like symptoms [27]. Furthermore, HSV1 encephalitis may also trigger AE mostly with anti-NMDAR antibodies [22]. However, post-herpetic AE seems to be a different etiopathogenic entity with a likely distinct genetic predisposition that could involve Toll-like receptor 3 [100].

Therefore, genetic predisposition to anti-NMDAR encephalitis remains undefined, and should ideally be investigated in large and diverse cohorts using general approaches such as GWAS, rather than preselected genes, in order to determine whether the genetic background of this disease is as heterogeneous as the acquired factors related to it.

## 5. Conclusions and Future Perspectives

The increasing knowledge about the pathophysiology of the immune system is providing new perspectives on the mechanism underlying autoimmune neurological disorders and offering novel candidates as biomarkers. However, the development of biomarkers in rare disorders, such as anti-NMDAR encephalitis, may be challenging due to the difficulties related to collecting large cohorts of patients to achieve solid conclusions. For these reasons, the majority of biomarkers validated in anti-NMDAR encephalitis are clinical or paraclinical, whereas most soluble biomarkers are still at the early phases of their development, with the notable exception of CSF IgG NMDAR antibodies that have been widely implemented in clinical practice.

Future investigations should consider the aforementioned limitations to decipher the pathophysiology of anti-NMDAR encephalitis and develop novel biomarkers to guide clinical decisions. In addition, international collaborative studies are required for recruiting large cohorts of patients with anti-NMDAR encephalitis in order to increase the strength and reliability of their results.

## Figures and Tables

**Table 1:** Diagnostic criteria for anti-NMDAR encephalitis.

Probable Anti-NMDAR Encephalitis
Diagnosis can be made when all three of the following criteria have been met: Rapid onset (less than 3 months) of at least four of the six following major groups of symptoms: Abnormal (psychiatric) behavior or cognitive dysfunction; Speech dysfunction (pressured speech, verbal reduction, mutism); Seizures; Movement disorder, dyskinesias, or rigidity/abnormal postures; Decreased level of consciousness; Autonomic dysfunction or central hypoventilation; At least one of the following laboratory study results: Abnormal EEG (focal or diffuse slow or disorganized activity, epileptic activity, or extreme delta brush); CSF with pleocytosis or oligoclonal bands. Reasonable exclusion of other disorders. Diagnosis can also be made in the presence of three of the above groups of symptoms accompanied by a systemic teratoma.
Definite anti-NMDAR encephalitis
Diagnosis can be made in the presence of one or more of the six major groups of symptoms and IgG GluN1 antibodies *, after reasonable exclusion of other disorders.

CSF, cerebrospinal fluid; EEG, electroencephalogram; IgG, immunoglobulin G; NMDAR, N-methyl-D-aspartate receptor. * Antibody testing should include testing of CSF. If only serum is available, confirmatory tests should be included (e.g., live neurons or tissue immunohistochemistry, in addition to cell-based assay).

**Table 2:** CSF soluble inflammatory molecules proposed as biomarkers of anti-NMDAR encephalitis.

Type of Biomarker	Findings	Molecule	References
Clinical activity	Acute phase	CXCL-13	[73,74,75,76,77,78]
		BAFF and APRIL	[79]
		IFN-γ	[74,78,80]
		TNF-α	[74,76,78,80]
		CXCL-10	[74,76,78]
		CCL-22	[77,78]
		IL-1β	[77,81]
		IL-6	[75,76,77,81,82]
		IL-7	[74,78]
		IL-10	[76,78]
		IL-17A	[74,75,77,81,82,83]
		NLRP3	[81]
		CD146	[84]
	Elevated for months after the acute phase	IFN-γ, TNF-α, CXCL-10, IL-7, IL-17A	[74]
	Relapses	CXCL-13	[73,76]
		CXCL-10	[76]
		IL-17A	[77]
	Clinical severity	CXCL-13	[76]
		CXCL-10	[76,78]
		CCL-22	[77,78]
		IL-6	[77,78,81,83]
		IL-10	[78]
		IL-17A	[81,83]
		CHI3L1	[85]
		OPN	[85]
		CD138	[80]
		CD40L	[83]
		PTX3	[83]
		sFas and sFasL	[86]
Inflammatory activity	CSF antibody titers	CXCL-13	[73]
	Pleocytosis	CXCL-13, CXCL-10	[74]
Treatment response	Limited response	IL-17A	[77]
Outcomes	Poor long-term outcomes	CXCL-13	[73]
		BAFF and APRIL	[79]
		CXCL-10	[76]
		IL-17A	[77]

APRIL, a proliferation-inducing ligand; BAFF, B-cell activating factor of the tumor necrosis factor family; CCL22, chemokine C-C motif ligand 22; CSF, cerebrospinal fluid; CHI3L1, chitinase-3-like 1; CXCL, C-X-C motif chemokine; sFas, soluble Fas; sFasL, soluble Fas ligand; HMGB1, high-mobility group box 1; IFN-γ, interferon γ; IL, interleukin; NLRP3, NOD-like receptor family, pyrin domain-containing 3; NMDAR, N-methyl-D-aspartate receptor; OPN, osteopontin; PTX3, pentraxin 3; TNF-α, tumor necrosis factor-α.
